# The Interaction of Agricultural Pesticides and Marginal Iodine Nutrition Status as a Cause of Autism Spectrum Disorders

**DOI:** 10.1289/ehp.11010

**Published:** 2008-04

**Authors:** Kevin M. Sullivan

**Affiliations:** Rollins School of Public Health Emory University Atlanta, Georgia E-mail: cdckms@sph.emory.edu

Roberts et al. (2007) recently reported on the results of their investigation into the relationship between agricultural pesticides and autism spectrum disorders (ASD) and found an association between organochlorines and ASD. One possible mechanism for this relationship is through thyroid disruption (Cheek et al. 1999). There is evidence to suggest that iodine deficiency might be associated with some of the increase in the reported prevalence/incidence of autism (Sullivan and Maberly 2004). For pregnant women who have a marginal iodine nutrition status, the disruption of the thyroid due to exposure to organochlorines could induce iodine deficiency and result in negative effects on the brain of the developing fetus. The U.S. iodine nutrition status has declined markedly over the last three decades, with the current iodine nutrition status among pregnant women being marginal (Caldwell et al. 2005; Hollowell et al. 1998). Because of the current iodine status of pregnant women, the Public Health Committee of the American Thyroid Association (2006) has recently recommended that all pregnant and lactating women take daily iodine supplements. It is interesting that the ASD case mothers tended to be older and more likely to be non-Hispanic white and non-Hispanic black than controls, which is consistent with a poorer iodine nutrition status in older women and in non-Hispanics in the United States (Caldwell et al. 2005; Hollowell et al. 1998).

Ensuring adequate iodine nutrition status of women, especially throughout pregnancy, is an extremely important public health goal. Given the negative effects of a number of environmental chemicals on the thyroid (Zoeller and Crofton 2000), it becomes increasingly important to ensure that all women have an adequate iodine intake and that the recommended approach to assuring adequate iodine nutrition is through a comprehensive iodized salt program (International Council for Control of Iodine Deficiency Disorders/United Nations Children’s Fund/World Health Organization 2001; Sullivan 2007).

## Editor’s note

In accordance with journal policy, Roberts et al. were asked whether they wanted to respond to this letter, but they chose not to do so.
